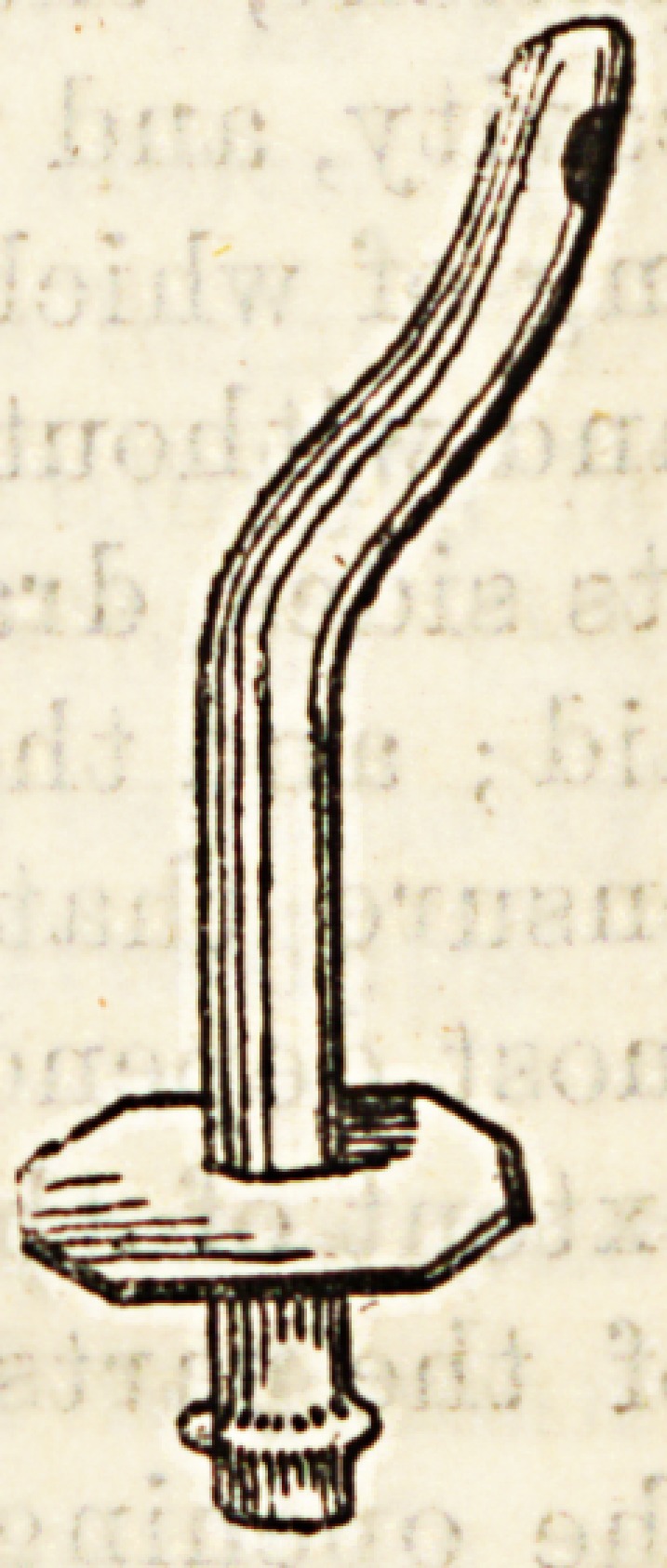# The Drainage of Wounds and Cavities

**Published:** 1894-01-27

**Authors:** 


### Medical Progress and Hospital Clinics.

[*The Editor will be glad to receive offers of co-operation and contributions from members of the profession. All letters should be addressed to* The Editor, The Lodge, Porchester Square, London, W.]

### LONDON HOSPITAL.

### The Drainage of Wounds and Cavities.

Amongst the principles of modern surgery few points are of greater importance than the maintenance of efficient drainage in wounds and cavities, in such a manner as to promote healing or the free escape of pus: ensuring primary union of wounds on the one hand, speedy closure of suppurating cavities on the other.

For this purpose in the greater number of cases some means has to be provided for conducting away from the wound or cavity the discharges that would hinder a quick return to health. The means taken for ensuring this result are suitable elastic pressure, with attention to position, and providing for exit of the discharges by means of suitable tubes or other bodies that, acting by capillary attraction, are the mechanical equivalent of tubes.

Several points have to be considered in drainage by position—the position of the bed and body of the patient, the position of his limb, of the wound or cavity, and the openings in them. A wound, the opening of which is in the lowest and most dependent part, and without any pouches or sinuses leading away from its sides, drains naturally and without any adventitious aid; and the aim of the surgeon is to, as far as possible, ensure that the openings of wounds shall be in the most dependent part, as far as is compatible with the extent of the disease and the anatomical arrangement of the parts. This is esteemed specially necessary in the opening of abscesses and other suppurating cavities, and if the primary incision cannot be made over the lowest level of the fluid, the same result is obtained by making, at the termination of the operation, a counter-opening for drainage purposes at the most dependent point.

Not only is attention paid to the position of openings, but to the position of the limb and patient after operation. A limb having in some part of its extent a suppurating or discharging site, is, if possible, so arranged in bed that the suppurating part is the lowest; for instance, a hand where suppuration is taking place, either in the palm or one of the fingers, is kept low, so that the pus may not be aided by the force of gravity in tracking up the tendon sheaths to the arm; so in empyema, and the patient is kept at first to as great an extent as is compatible with comfort on the affected side, so that the pus may be guided to what is then the lowest part of the chest cavity, near which position the opening for its evacuation is made. In suppuration about the lower extremities the same use is made of position by raising the head of the bed a few inches on blocks, by which the tendency of fluid to track up the limb is to a certain extent counteracted.

Coming now to the more actual means of removing fluid, they all consist essentially of the careful combination of well-regulated pressure with a system of more or less rigid tubes for the exit of discharges. The tubes most in use are the ordinary rubber ones, made either of red or black rubber, of various sizes, and perforated at set intervals for the admission of discharge. Another variety of rubber tube is also used, consisting of a tube formed by a spiral of red rubber; this gives very perfect drainage, but has the disadvantage of collapsing very easily under pressure, besides being more expensive than the ordinary rubber tubing.

A few points have to be considered in the use of rubber drainage tubes, the most useful and widely used of all methods of drainage. The calibre of the tube has to be proportionate to the size of the cavity and the duration of its use. A tube must be large enough to give free exit to discharge, but not so large

Fig. 1.

Fig. 2.

as to be acting as an irritant; for however aseptic they may be, a tube is still a foreign body in a wound, and so tends to keep up discharge; nor, as a rule, is a tube chosen so large as to be a means of entrance for air into the interior of a cavity. To be perfect, it should be just large enough to allow all discharge to escape, but to let in little air. A tube is cut long enough to reach so far in that there shall be no undrained pocket beyond the innermost end, but not so long that it bends upon itself, as a bend stops the flow of fluid along it. The end outside the wound is, as a rule, cut off flush, or nearly so, with the skin; the reason for so doing is that the pressure of dressings may not push it further into the wound than is intended, either bending it and stopping the flow of fluid, or pushing it injuriously against the growing and granulating tissues in contact with its innermost end. To prevent the tube from slipping into the opening and getting lost, either a safety pin is fastened through the free end, and as it lies flat on the skin at right angles to the incision, prevents it slipping in, or a thread of silk is stitched through the outer end, and the silk fastened to the skin at some distance from the wound with adhesive plaster, or left among the dressings. In those cases where there would be great difficulty in reinserting the tube, should it accidentally come out, it is fastened by a stitch to the margin of the skin. The first of these plans is the best, the silk, if used for any length of time, becoming saturated with pus, with the inevitable liability to become septic. Where a tube is inserted into a large cavity, such as the pleura in opening an empyema, it is split for an inch or an inch and a half down, the two ends turned out and kept at right angles to the rest of the tube by means of a large safety or hardclip pin (Fig. 1), or the tube is passed through a hole in a piece of thick sheet gutta percha, and the split ends fastened to it by means of a couple of stitches of silver wire. (Fig. 2).

The time a tube is left in position depends on the nature of the condition for which it is being used. As a general rule, if it requires reinsertion it is not removed till granulations have sprung up round it, so that when taken out a patent sinus is left, into which the cleansed tube can be easily inserted with a gentle corkscrew action, and the reinsertion is rendered easier by having the end of the tube bevelled off to a point, not cut square and at right angles to its long axis. When used for operation wounds the tubes can be removed or shortened much sooner than when used for suppurating cavities, but in no case are they left in so long that granulations grow into the openings, rendering removal painful and damaging the growing tissues.

The necessary points in the drainage of operation wounds are the removal of any blood that may collect in the wound, and excess of the inevitable serous outflow from the cut tissues. The necessity for the free removal of serous fluid is only of fairly recent recognition, reabsorption of serum under pressure being attended in the first place with a rise of temperature, it may be up to 103 degrees or 104 degrees; and, secondarily, if air be admitted by suppuration of the retained discharge.

The necessity for the removal of serum is greatest during the first twenty-four hours, and to this end great attention is paid to the primary position and size of the drainage tubes. They are, as a rule, removed or shortened at the first dressing, on the 5th to the 10th day from the operation, when primary union has taken place through the greater extent of the wound.

The great guide as to whether drainage in a given case is efficient is the temperature. A rise of temperature the first two days after operation usually indicates absorption of serous discharge, and therefore ineffectual drainage, a point that is then remedied by clearing a tube perhaps blocked with clot, or removal of a suture relieving tension on the retained fluid. Rise of temperature later on more often indicates that suppuration is taking place, or, in the case of a suppurating cavity, that the tension of the retained pus has risen to such a point that reabsorption is taking place; in any case the course pursued is to look and see, and by freer openings or use of antiseptics to remedy the evil.

For use in the drainage of the different cavities of the body tubes of special form are used. A form of tube, known as Perrin's, is used for draining the bladder through a suprapubic wound, but is also extremely useful for draining deep suppurating cavities that require washing out.

In form it consists of two red rubber tubes like two large long red rubber catheters with very large eyes, and varying in diameter from a quarter to half an inch, and fastened back to back, so that the two eyes point in opposite directions.

When put into the bladder through the suprapubic wound one tube is cut off, so that about an inch projects above the skin, and is surrounded by the dressings, the other end comes through the dressings, and is conducted into some antiseptic in a vessel alongside the patient, and through this tube the urine drains from the bladder into the vessel. In washing out the bladder fluid is injected into the shorter tube and comes out through the longer, washing away any clots, &c., that may be present.

These tubes can easily be improvised by sewing together two of the largest red rubber catheters through the eyes, so that the eyes face in directly opposite directions, cutting one catheter as short as may be required, and if the other is not long enough, connecting it with a piece of rubber tubing, the end of which can be placed in some antiseptic fluid.

Another tube that is used for the drainage of the peritoneal cavity is the well-known Keith's tube, made of glass, of various sizes as regards length and diameter. They consist of a piece of glass tubing about half an inch in diameter, open at both ends, and towards the innermost end perforated with numerous small holes; at the outer end, a little from its termination, is a flange.

The tube having been put through the abdominal incision, the deep end usually being in the pouch of Douglas, the flange just reaches to the level of the skin.

A piece of sheet india-rubber about twelve inches square is taken, a slit cut in its centre, and is put over the tube so that the flange and upper end comes through the slit like a button through a button-hole, the tube being firmly encircled below the flange by the elastic rubber. The end of the tube being covered with a sterilised sponge the edges of the rubber are turned over it; and the rest of the abdominal incision being closed by sutures, discharge can only find its way through the tube into the sponge, being prevented by the sheet rubber from penetrating the rest of the dressings. When the patient lies on her back in bed the pouch of Douglas is the lowest part of the abdominal cavity, and therefore in it such fluid as may be present in the abdominal cavity tends to collect, and is removed through the Keith's tube by suction with a clean glass syringe attached to a new sterilised rubber catheter or rubber tube, the end of which is passed through the tube to the bottom of the pouch and the ocntents withdrawn into the syringe.

Another special form of tube is that used for draining the bladder through the perineum. It is usually made of vulcanite curved so as to fit easily through the wound into the bladder, having a flange with holes for tapes, which passing up in front and behind can be attached to a belt, keeping the tube in position. At the extreme free end of the tube is a smaller flange, to which a rubber tube can be attached, and led to a vessel containing some antiseptic fluid, into which the contents of the bladder can drain.

For the drainage of small wounds, especially about the face, where the mark left by the track of a drainage tube would be a disfigurement and where the discharge is slight, a few strands of horsehair, properly cleaned in an alkali and sterilised in carbolic acid solution, are used. For small wounds with slight discharge they make a useful and efficient drain, which can be diminished by withdrawing a few strands of the hair at a time without removing the whole. Instead of the horsehair, catgut, or gutta percha tissue are sometimes used; of the two the gutta percha is the most efficient. A small strip is rolled up so as to form a small tube, and when the discharge is slight answers well. Catgut is more apt to be saturated with the discharge, and does not drain so well.

In all cases carefully regulated pressure to obliterate the discharging cavity is used in conjunction with the now well-known principles of antiseptic surgery to prevent or lessen the decomposition of discharges, and promote the speedy union of wounds.

## Figures and Tables

**Fig. 1. f1:**
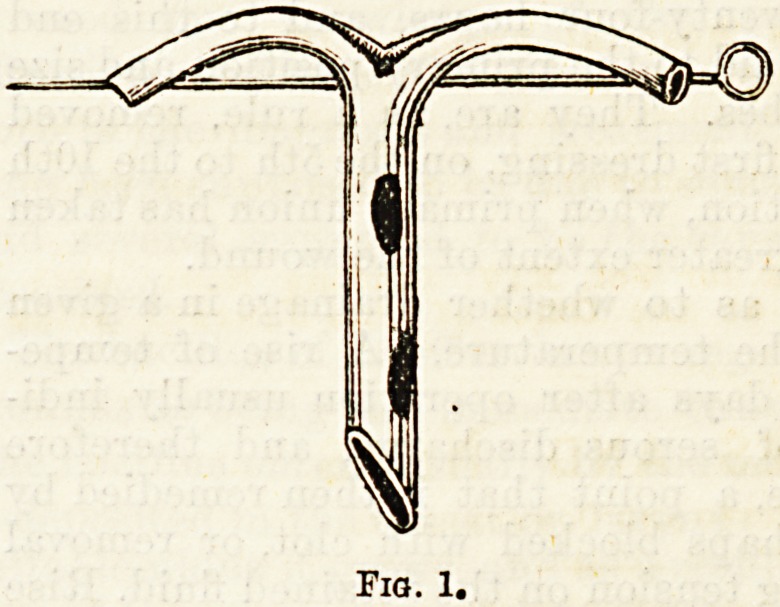


**Fig. 2. f2:**
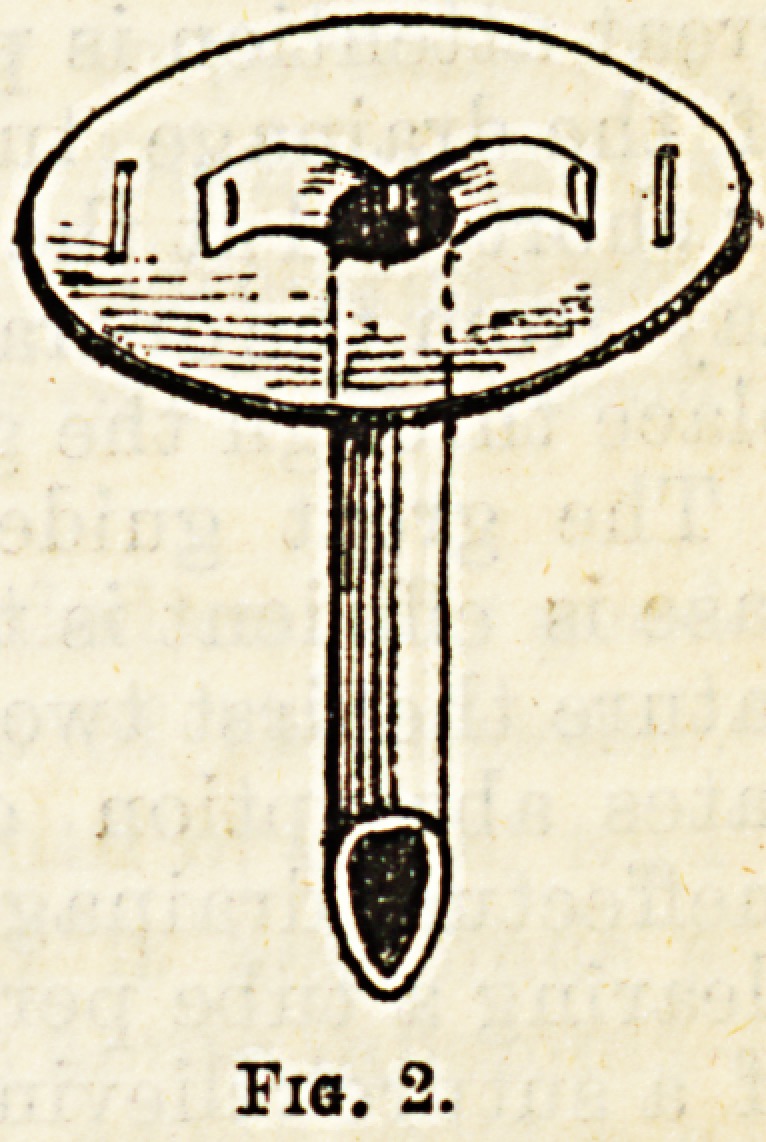


**Figure f3:**
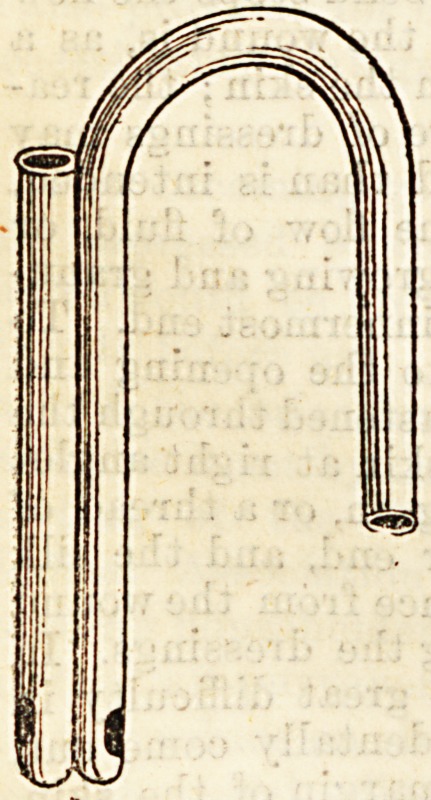


**Figure f4:**
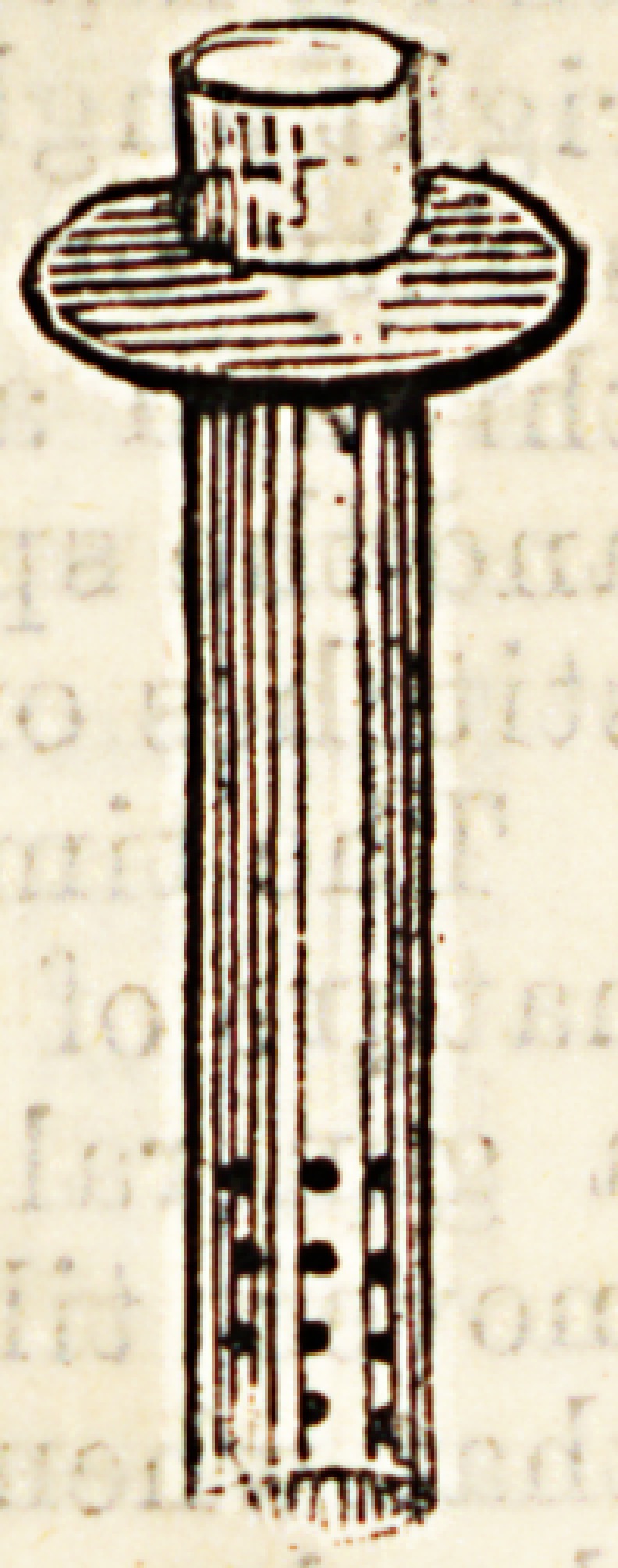


**Figure f5:**